# Exploration and Analysis of Cochlear Implant Content on Social Media

**DOI:** 10.7759/cureus.45801

**Published:** 2023-09-22

**Authors:** Sharon J Feng, Michelle Yu, Stephen Leong, Alexander Chern

**Affiliations:** 1 Department of Otolaryngology–Head & Neck Surgery, Columbia University Irving Medical Center/New York-Presbyterian Hospital, New York, USA; 2 Vagelos College of Physicians and Surgeons, Columbia University, New York, USA; 3 Department of Otolaryngology–Head & Neck Surgery, Weill Cornell Medical College/New York-Presbyterian Hospital, New York, USA; 4 Department of Otolaryngology–Head & Neck Surgery, University of Washington, Seattle, USA

**Keywords:** hearing loss, tiktok, cochlear implant, social media, otology, otolaryngology

## Abstract

Introduction: Social media has an ever-growing presence in patients’ lives, particularly with the dissemination of both useful and misleading information. We performed an exploration and analysis of content pertaining to cochlear implantation on a popular social media platform.

Methods: “Cochlear implant” (CI) was queried on TikTok in early October 2022 in a cross-sectional analysis. The 100 top videos were collected. Non-English and duplicate videos were excluded. Two independent researchers used the Global Quality Scale (GQS) and modified DISCERN tool to score videos; higher ratings indicate increased quality and reliability. Demographic data were also recorded.

Results: Of the 100 videos assessed, 95 met the inclusion criteria. The average video had 2.36 million views, 328 thousand likes, and a duration of 36.1 seconds. The mean GQS was 1.91 (standard deviation (SD) 0.85), and the modified DISCERN score was 1.48 (SD 1.20). Posters were predominantly laypersons (93.7%), including CI users or parents of pediatric CI users. No videos featured audiologists or otolaryngologists. Nearly half of the videos (48.4%) discussed the experience of a patient or parent of a pediatric patient with CIs, and 24.2% were aimed at directly educating the viewers about CIs or the deaf community.

Conclusions: Most videos featured CI users or families of pediatric CI users and detailed specific patient experiences. The deaf and CI communities have strong social media presences. Most videos had limited quality and reliability, measured via the GQS and modified DISCERN tool, and no videos featured hearing healthcare professionals, highlighting opportunities for clinicians to use the platform as a patient resource.

## Introduction

According to the National Institute on Deafness and Other Communication Disorders (NIDCD), over 700,000 individuals worldwide have received a cochlear implant (CI). In the USA alone, about 118,100 devices have been implanted in adults and 65,000 in children [1]. It is widely known that the CI community has used social media for support, advocacy, rehabilitation information, research endeavors, and sharing of personal experiences [2]. However, social media continues to rapidly evolve, and as it evolves, the content that is created and shared may also evolve. CI content on TikTok (ByteDance Ltd., Beijing), an expanding social media platform with over one billion active users, has not been thoroughly investigated.

The ease of consuming and creating content on social media has only grown in recent years. Patients have also begun to utilize social media for medical information; one recent 2021 survey found that approximately one in 10 polled Americans turned to social media when looking for reliable health information [3]. Popular social media platforms, such as TikTok and Twitter, specialize in short-form content and do not require the user to sign up for an account to view content. This convenience factor means that medical content can reach a wider audience than ever before; notably, the hashtag “cochlearimplant” has over 1.1 billion views on TikTok as of May 2023. Content creators can utilize these platforms to share information in real time, sometimes even live.

There is a growing attention in the literature on the importance of social media within otolaryngology and the CI community. Saxena et al. (2015) found that the CI online community is highly decentralized, with no single platform dominating the conversation [2]; notably, the landscape of social media is constantly evolving, and TikTok was launched after the publication of this article. In a more recent study, Rossi et al.* *(2023) analyzed over 500 posts on TikTok and noted that most posters were either patients or family members of patients [4]. However, no prior research has examined the quality and reliability of social media content pertaining to CIs. In this study, we aim to assess the landscape of CI content on TikTok to better understand how healthcare professionals can use social media to understand the patient experience and inform patient education.

## Materials and methods

A cross-sectional analysis was conducted on October 9, 2022, where “cochlear implant” was queried on TikTok and the top 100 videos from that day were selected for analysis. Non-English and duplicate videos were excluded, resulting in 95 videos included in the final analysis. Demographic data collection included video views, duration, date posted, hashtag, poster category (i.e., layperson, physician, audiologist, corporate account, and others), age group of the patient featured (adult, pediatrics, and others), use of American Sign Language (ASL) in the video, category of video (i.e., patient experience, educational, skit, TikTok sound trend, inspirational, etc.), and tone toward CIs (neutral, positive, or negative) [4-6].

**Figure 1:**
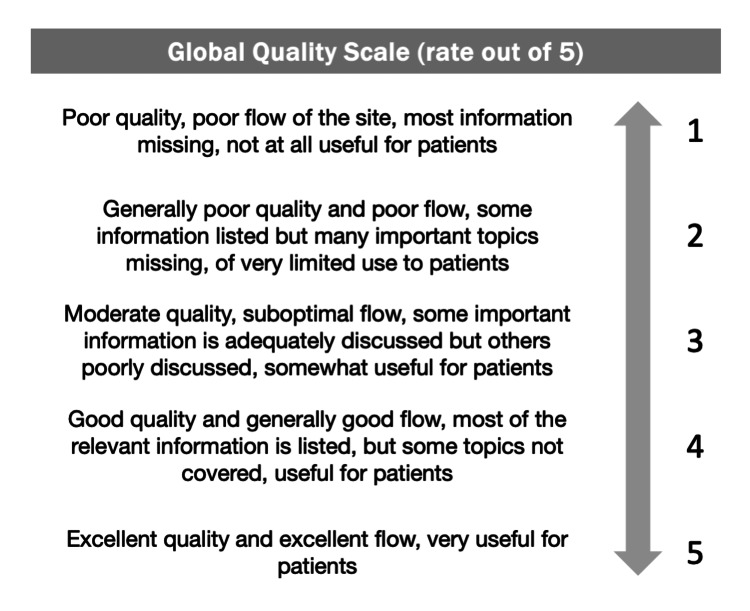
Global Quality Scale (GQS) grading criteria

Two independent researchers utilized the Global Quality Scale (GQS) and modified DISCERN tool to score each video (Figure 1, Table 1) [7,8]. The GQS score is a single scale from 1 to 5, where a score of 1 represents information that is of poor quality and has no use to patients. A score of 5 represents information that has excellent quality and is very useful to patients. The modified DISCERN tool evaluates the quality of health content through five categories, which are scored on a binary scale: 1 for “yes” and 0 for “no.” The five categories are as follows: 1) clear aims or goals, 2) use of reliable citations or sources, 3) lack of bias, 4) explicit reference to other sources for further exploration, and 5) discussion of areas of uncertainty. Scores from each of the five categories are summed up to calculate the final quality metric. 

**Table 1:** Modified DISCERN tool scoring criteria

Modified DISCERN score (rate each question 0 = no, 1 = yes for total score of 5)
Are the aims clear?
Are reliable information sources used?
Is the information balanced and unbiased?
Are additional sources listed for patients?
Are areas of uncertainty stated?

## Results

The top 100 TikTok videos on CIs were on average 36.1 seconds in length, had 2.36 million views, and had 328 thousand likes. Notably, nearly half of videos (48.4%) focused on the firsthand experiences of patients or parents of pediatric patients who have received CIs. A proportion (41.1%) of videos contained ASL. Another proportion (24.2%) were aimed at directly educating viewers about CIs or the deaf community. Meanwhile, 72.6% of the videos had a neutral tone, 16.8% of videos had a positive tone, and 10.5% of videos had a negative tone. The characteristics of the top 20 most liked videos in our dataset are displayed in Table 2.

**Table 2:** Characteristics of the top 20 most-liked TikTok videos within our dataset ASL: American Sign Language

User	Views	Duration	Likes	Date posted	Poster	Age	ASL	Video type	Tone
deal_family	20,400,000	0:26	2,800,000	10/21/21	Layperson	Adolescent	Y	Skit	Neutral
brittneynolte	18,200,000	0:42	2,500,000	1/23/22	Layperson	Pediatrics	Y	Patient experience	Neutral
emstewie17	13,400,000	0:06	1,300,000	9/22/22	Layperson	Pediatrics	N	Patient experience	Neutral
sarahtessax	12,600,000	0:29	2,600,000	3/19/22	Layperson	Adolescent	Y	Skit	Negative
christykeanecan	12,500,000	0:15	1,600,000	2/6/20	Layperson	Pediatrics	N	Educational	Neutral
babyfaceniko	10,600,000	1:08	1,900,000	6/14/22	Layperson	Pediatrics	N	Patient experience	Neutral
deal_family	10,100,000	0:38	2,000,000	12/5/20	Layperson	Adolescent	Y	Educational	Neutral
cristalallure	8,700,000	0:30	1,300,000	12/2/21	Layperson	Pediatrics	Y	Patient experience	Positive
babyfaceniko	8,400,000	0:10	892,700	6/9/22	Layperson	Pediatrics	Y	Patient experience	Positive
cargo_shorts_dad	7,700,000	0:35	1,400,000	5/7/21	Layperson	Pediatrics	Y	Educational	Neutral
milathehearingdog	7,600,000	2:59	1,600,000	12/30/21	Layperson	Adult	N	Patient experience	Positive
deal_family	5,400,000	0:30	679,000	2/9/21	Layperson	Pediatrics	N	Skit	Neutral
thebarnesbunch	4,500,000	0:25	644,300	2/7/21	Layperson	Pediatrics	N	Patient experience	Neutral
deal_family	4,400,000	0:29	411,700	11/27/21	Layperson	Adolescent	N	Skit	Neutral
christina_pax	4,300,000	1:01	621,200	12/29/21	Layperson	Pediatrics	Y	Patient experience	Neutral
scarlet_may.1	4,100,000	0:49	985,900	1/26/21	Layperson	Adolescent	Y	Skit	Neutral
deal_family	3,400,000	0:21	263,200	2/23/22	Layperson	Adolescent	N	Skit	Neutral
brittneynolte	3,4000,00	0:29	555,100	2/13/21	Layperson	Pediatrics	Y	Patient experience	Neutral
ashleigh2864	3,300,000	0:22	506,000	5/2/21	Layperson	Adult	N	Patient experience	Positive
scarlet_may.1	3,200,000	0:14	425,400	7/7/22	Layperson	Adolescent	Y	Educational	Positive

**Figure 2:**
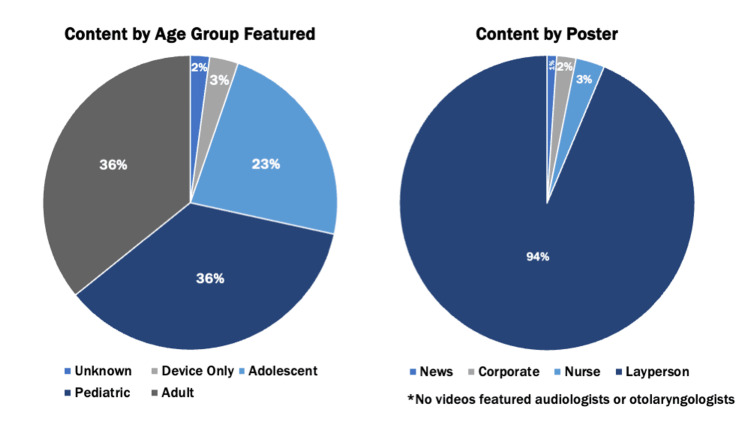
Percentage of TikTok videos sorted by age of the subject featured (left) or by the creator (right)

The videos featured a mix of patients in the pediatric (36%), adolescent (23%), and adult (36%) age ranges (Figure 2). Three percent of the videos depicted devices only and did not mention the age of the target user demographic. The posters were predominantly laypersons (93.7%), including CI users or parents of pediatric CI users. A very small percentage of videos were posted by creators who were nurses (3.2%), corporate accounts (2.1%), or news outlets (1.1%). No videos featured audiologists or otolaryngologists.

**Figure 3:**
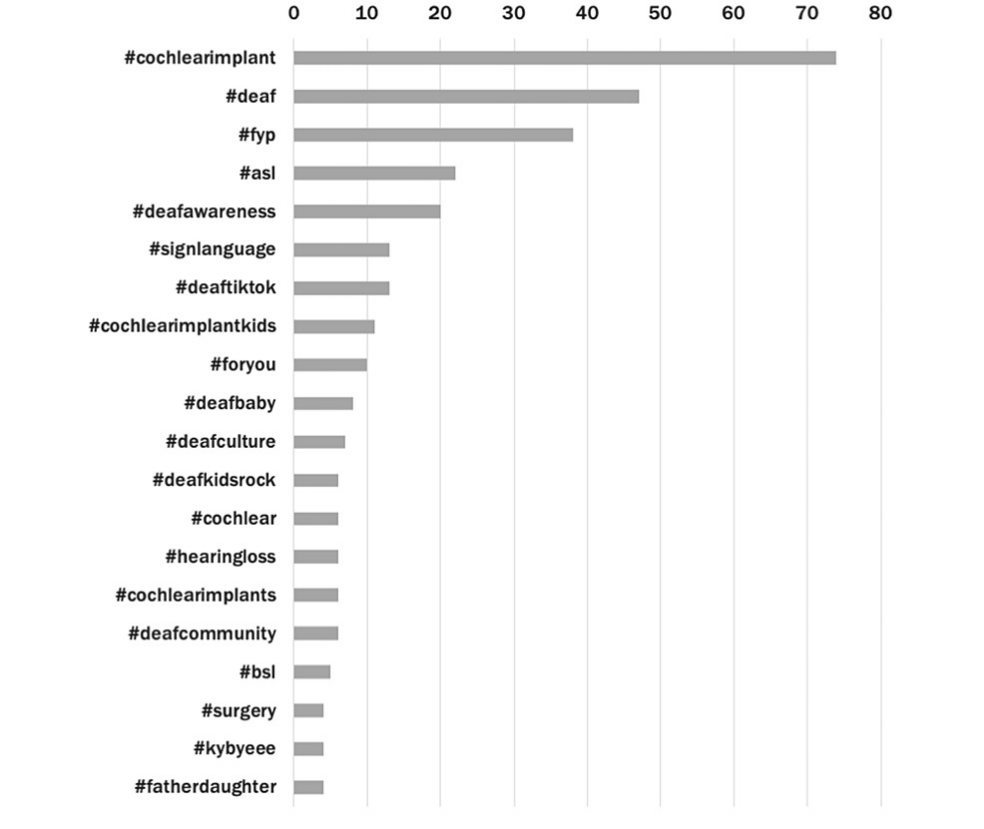
Common hashtags featured in the “cochlear implant” dataset Data were taken from the top 100 "cochlear implant" TikTok's common hashtags.

Top hashtags (>10 videos) included #cochlearimplant, #deaf, #fyp, #asl, #deafawareness, #signlanguage, #deaftiktok, and #cochlearimplantkids (Figure 3). In total, the top 100 TikTok videos contained 182 unique hashtags, the vast majority of which were only used once within our dataset. Hashtags mainly contained themes relating to CIs, deafness and deaf culture, family, and children. Some hashtags were clearly targeting the algorithm in attempts to increase viewership (i.e., #fyp, #foryourpage, and #viral).

**Figure 4:**
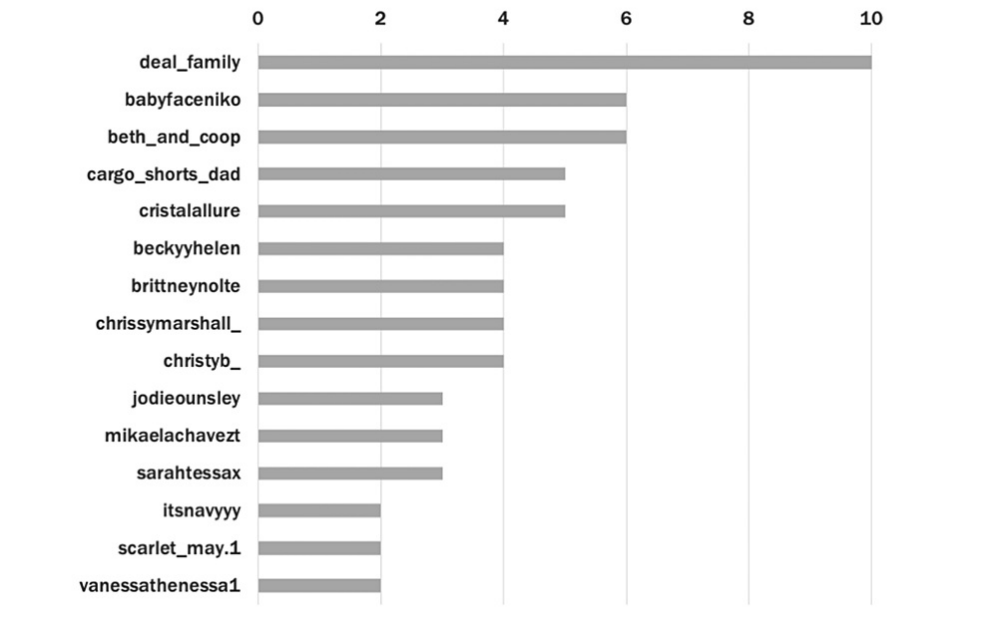
Most prolific creators within the “cochlear implant” dataset Data taken from the top 100 "cochlear implant" TikTok's most common users.

The top 100 TikTok videos were created by 47 users, with the top 10 creators accounting for more than half (51.0%) of the content in our dataset (Figure 4). The most prolific user, “deal_family,” accounted for 10% of the videos, produced by TikTok creators Brandon and Megan Deal. On their multiple social media platforms, Brandon and Megan Deal document their journey parenting their two daughters, one of whom is deaf and has a CI. Their content ranges from fun, lighthearted vlogs to educational short-form content about hearing loss and CIs; this content is reflective of many other videos on this platform.

The videos contained in this dataset were independently scored by two researchers using the GQS and modified DISCERN quality scales. The mean combined GQS score was 1.91 out of a possible score of 5 (standard deviation (SD) 0.85), suggesting that the content of videos is of generally poor quality with limited usefulness to patients (Table 3) [7]. The mean GQS score from reviewer 1 was 1.95 (SD 0.82); the mean score from reviewer 2 was 1.86 (SD 0.87). The mean combined DISCERN score was 1.48 out of a possible score of 5 (standard deviation of 1.20), consistent with the GQS ratings [7]. The mean DISCERN score from reviewer 1 was 1.48 (SD 1.14); the mean score from reviewer 2 was 1.47 (SD 1.27).

**Table 3:** GQS and modified DISCERN tool scores GQS: Global Quality Scale; SD: standard deviation

	Top 100 TikTok videos for “cochlear implant”
Mean GQS score	1.91 (SD 0.85)
Mean modified DISCERN score	1.48 (SD 1.20)

## Discussion

The overall quality scores for the top 100 CI TikTok videos were low when analyzed using the GQS and DISCERN scoring systems. Notably, no videos were created by otolaryngologists or audiologists (i.e., the clinicians who work most closely with CI users), even though nearly a quarter of videos were aimed at patient education. This disparity represents an opportunity for healthcare providers to reach patients and their family members using a popular, free-of-cost, and accessible social media platform. Increasing accessibility to basic medical information is especially important; according to a recent Gallup poll, 38% of Americans in 2022 skipped or delayed medical care due to healthcare costs [9]. Greater public awareness of common medical conditions may empower patients and their families to make informed decisions when seeking medical care, particularly in the context of cochlear implantation.

CI content on TikTok was predominately created by patients or family members of CI users and highlighted patient experiences. Creators discussed personal experiences with diagnoses of hearing loss, pros and cons of implantation, expectations versus reality of CIs, and whether they would recommend CIs to others. The lower GQS and modified DISCERN scores may be explained by the fact that patient anecdotes typically do not cite literature or other credible sources. Thus, the issue may stem from the metrics themselves and not the content under study, as these grading scales are not designed to capture a single patient’s experience or evaluate mainstream media. In fact, social media sites give patients a platform to provide crucial insights into the everyday experiences of living with CIs. This, in turn, increases the visibility of CIs within both the deaf community and the broader public. As a whole, the CI community on TikTok is focused on creating a safe and inclusive education space; content creators are predominantly patients or families of patients aiming to 1) educate other families who are considering or have cochlear implants and 2) inform the general public about the experience of living with CIs. 

Social media provides many other tangible benefits to patients. Importantly, increased representation and discussion of hearing loss and CI on social media serves to normalize and destigmatize hearing loss [10]. Media portrayals and public perceptions of hearing loss are often limited to negative stereotyping, and the first-person perspectives of creators with hearing loss offer a direct means of countering these stereotypes [11-13]. Moreover, social media can be a source of convenient and easily digestible health information for individuals who may not otherwise seek out medical care [3,14]. In this way, social media can effectively lower barriers to health education and medical information, while simultaneously normalizing and destigmatizing hearing loss [10].

Notably, eight out of 10 Internet searches in the USA are related to health information, which underscores the intricate link between social media and healthcare [15]. It is thus of utmost importance that physicians acknowledge the critical role of social media and utilize it to promote well-being and combat misinformation. In fact, social media may have a mutually beneficial role for physicians and their patients. Patients learn about the physician’s role in their health maintenance, and physicians earn the trust of patients who may not normally seek their services. Importantly, professional guidelines and recommendations are available for practitioners who are interested in building a social media presence [15]. Organizations, such as the National Association of the Deaf, provide recommendations that can be utilized to produce accessible and respectful content on hearing loss and CI [16].

This study has several limitations related to the scope and metrics used to assess the quality of short-form content. First, the scope of our study was limited to the top 100 English language videos for the search “cochlear implant” in early October 2022, which captures only a snapshot of the content on the platform. A sample taken on another day, by another user, and in a different language could produce entirely different results than those obtained in this study. In addition, the metrics we used to assess the video quality were originally created to evaluate content produced by professionals for the general public. The vast majority of short-form video content relating to cochlear implantation is generated by the general public. Ultimately, patient experiences, by their nature, are difficult to objectively evaluate. The short video format also, to some extent, limits the ability of creators to provide comprehensive perspectives on living with CIs; other sites, such as YouTube, may allow creators to share more detailed perspectives. Our findings highlight the need for the development of validated metrics to more appropriately capture patient-generated content. Future work will turn to other topics within otolaryngology and cover additional platforms commonly utilized by patients and their families, such as Instagram, Twitter, Facebook, and YouTube.

## Conclusions

In this study, we queried “cochlear implant” on TikTok and evaluated 100 videos featuring CI users or families of pediatric CI users. Most of these videos detailed specific patient experiences. Not a single video in our dataset featured hearing healthcare professionals, highlighting opportunities for clinicians to use the platform as a patient resource. Most videos had limited quality and reliability measured via the GQS and DISCERN scoring systems. Current metrics undervalue shared patient experiences, which underscores the need for better methodologies to analyze patient-generated content. The deaf and CI communities maintain a strong social media presence, and this can ultimately be leveraged to increase awareness regarding CIs and improve access to information on hearing loss.

## References

[1] Cochlear Implants (2023). Cochlear implants. Disorders.

[2] Saxena RC, Lehmann AE, Hight AE, Darrow K, Remenschneider A, Kozin ED, Lee DJ (2015). Social media utilization in the cochlear implant community. J Am Acad Audiol.

[3] (2023). 1 In 10 Americans turn to social media for health information, new survey shows. https://www.forbes.com/sites/debgordon/2021/10/06/1-in-10-americans-turn-to-social-media-for-health-information-new-survey-shows/.

[4] Rossi NA, Devarajan K, Chokshi SN (2023). Social media depictions of cochlear implants: an Instagram and TikTok analysis. Otol Neurotol.

[5] Wu J, Trahair E, Happ M, Swartz J (2023). TikTok, #IUD, and user experience with intrauterine devices reported on social media. Obstet Gynecol.

[6] Parisi L, Mulargia S, Comunello F (2023). Exploring the vaccine conversation on TikTok in Italy: beyond classic vaccine stances. BMC Public Health.

[7] Uzun O (2023). Assessment of reliability and quality of videos on medial epicondylitis shared on YouTube. Cureus.

[8] Charnock D, Shepperd S, Needham G, Gann R (1999). DISCERN: an instrument for judging the quality of written consumer health information on treatment choices. J Epidemiol Community Health.

[9] (2023). Record high in U.S. put off medical care due to cost in 2022. Put Off Medical Care Due to Cost in.

[10] (2023). As more deaf people are seen on tv, others want to be heard. https://www.nytimes.com/2021/01/27/arts/television/deaf-tv-representation.html.

[11] Denham MW, Chern A (2022). Giving children with deafness a cape: amplifying diverse portrayals of hearing loss in media. Ear Nose Throat J.

[12] Ruusuvuori JE, Aaltonen T, Koskela I, Ranta J, Lonka E, Salmenlinna I, Laakso M (2021). Studies on stigma regarding hearing impairment and hearing aid use among adults of working age: a scoping review. Disabil Rehabil.

[13] Foss KA (2014). (De)stigmatizing the silent epidemic: representations of hearing loss in entertainment television. Health Commun.

[14] Chen J, Wang Y (2021). Social media use for health purposes: systematic review. J Med Internet Res.

[15] Ventola CL (2014). Social media and health care professionals: benefits, risks, and best practices. P T.

[16] (2023). Guidelines for media portrayal of the deaf community. https://www.nad.org/about-us/position-statements/guidelines-for-media-portrayal-of-the-deaf-community/..

